# *Schistosoma japonicum* Infection in Treg-Specific USP21 Knockout Mice

**DOI:** 10.1155/2021/6613162

**Published:** 2021-02-09

**Authors:** Youxiang Zhang, De-Hui Xiong, Yangyang Li, Guina Xu, Baoxin Zhang, Yang Liu, Shan Zhang, Qing Huang, Simin Chen, Fansheng Zeng, Jingyi Guo, Bin Li, Zhiqiang Qin, Zuping Zhang

**Affiliations:** ^1^Department of Pathogen Biology, School of Basic Medical Science, Central South University, Changsha 410078, China; ^2^Molecular Biology Research Center, School of Life Science, Central South University, 110 Xiangya Road, Changsha, Hunan 410078, China; ^3^Department of Microbiology and Immunology, Shanghai Institute of Immunology, Shanghai Jiao Tong University School of Medicine, Shanghai 200025, China; ^4^Key Laboratory of Control Technique for Schistosoma and Pathogen Infection for Dongting Lake Region, Yiyang Medical College, Yiyang 413002, China; ^5^Department of Epidemic Prevention, Armed Police Hospital of Hunan Province, Changsha, 410008 Hunan, China; ^6^Sichuan Center for Disease Control and Prevention, Chengdu, 610041 Sichuan, China; ^7^Key Laboratory of Parasite and Vector Biology, Ministry of Health; National Institute of Parasitic Diseases, Chinese Center for Disease Control and Prevention, Chinese Center for Tropical Diseases Research, Shanghai 200025, China

## Abstract

The E3 deubiquitinating enzyme ubiquitin-specific proteolytic enzyme 21 (USP21) plays vital roles in physiological activities and is required for Treg-cell-mediated immune tolerance. Using a murine model infected with *Schistosoma japonicum*, we observed that there were more cercariae developed into adults and more eggs deposited in the livers of the USP21^fl/fl^FOXP3^Cre^ (KO) mice. However, immunohistochemistry showed that the degree of egg granuloma formation and liver fibrosis was reduced. In USP21^fl/fl^FOXP3^Cre^ mice, levels of IFN-gamma, IL-4, anti-soluble egg antigen (SEA) IgG and anti-soluble worm antigen preparation (SWAP) IgG increased in blood, as determined using ELISAs and multiplex fluorescent microsphere immunoassays, while the levels of IL-10, lL-17A, IL-23, IL-9, and anti-SEA IgM decreased. In addition, the levels of the USP21 protein and mRNA in the liver and spleen of KO mice decreased. We further observed increased Th1 responses amplified by Tregs (regulatory T cells) and compromised Th17 responses, which alleviated the liver immunopathology. We speculated that these changes were related to polarization of Th1-like Tregs. Our results revealed the roles of USP21 in Treg-cell-mediated regulation of immune interactions between *Schistosoma* and its host. USP21 may have potential for regulating hepatic fibrosis in patients with schistosomiasis.

## 1. Introduction

Schistosomiasis is an acute and chronic parasitic disease caused by blood flukes (trematode worms) of the genus *Schistosoma*. According to the WHO Report 2017, schistosomiasis transmission was reported by 78 countries, and at least 220.8 million people required preventive treatment in 2017. Schistosomiasis affects almost 240 million people worldwide, and more than 700 million people live in endemic areas, mostly in sub-Saharan Africa [1]. People become infected when the larval form of the parasite penetrates the skin (during contacting with infested water) after being released from freshwater snails. The larvae develop into adult schistosomes inside the body. Adult worms live in the blood vessels, where the females release eggs. Some of the eggs are passed out of the body in the feces or urine to continue the parasite lifecycle. Other eggs are trapped in body tissues, causing immune reactions and progressive damage to organs [2].

The immune system of infected hosts encounters parasites in several stages of its life cycle: penetrating cercariae, migrating schistosomula, adult worms, and eggs produced by adult worm pairs. Parasites in the developmental stages express hundreds of antigenic moieties, many of which stimulate strong humoral and cellular immune responses [3]. Some of these responses continue to increase during chronic infection, and others are substantially reduced [4]. Observations in experimental mouse models of infection have elucidated the mechanisms governing the development and regulation of the pathogenic immune response in schistosomiasis [5]. Some factors, including IL-10, Tregs, B cells, antibodies, T cell anergy, macrophages, and microRNAs, are related to the pathogenic immune response in schistosomiasis [6]. In addition, several studies have suggested that Th17 cells are involved in the pathogenesis of both *Schistosoma mansoni* and *Schistosoma japonicum* infection [7].

Tregs have an immunosuppressive capacity that is essential for maintaining immune homeostasis and controlling immune tolerance. The induction of Tregs inhibits the development of granuloma pathology [8]. FOXP3 is a crucial factor involved in producing the immunosuppressive phenotype of Tregs. Unstable FOXP3 makes the Treg-cell phenotype unstable and may bias the generation of different T helper cell phenotypes under different inflammatory conditions [9]. Furthermore, studies conducted with a conditional knockout mouse model suggested that USP21 stabilized the expression of the FOXP3 protein in Tregs by deubiquitination, thereby regulating the function of Tregs. A Th1-like sputum cell response, as observed with USP21-deficient Tregs, preferentially transforms into a Th2 cell-like phenotype in a host with a severe Th2-type disorder, whereas under arthritic conditions, Tregs may lose FOXP3 expression and transform into cells with a Th17 cell-like phenotype [10]. More importantly, FOXP3, GATA3, and USP21 are closely associated with Tregs. In Tregs, FOXP3 binds to the promoter region of USP21 and activates its transcription, and USP21 interacts with GATA3 and deubiquitinates it, thus inhibiting its degradation by the proteasome and maintaining its stability.

Moreover, GATA3 regulates the function of T cells in the inflammatory response by stabilizing the expression of FOXP3. Thus, in Tregs, the three proteins described above form a positive feedback pathway. Furthermore, studies conducted with conditional knockout mouse models suggested that USP21 regulated the expression of the FOXP3 protein in Tregs by deubiquitination, thereby regulating the function of Tregs [9]. In antiviral responses, USP21 binds to and deubiquitinates RIG-I in the cytoplasm to exert an immunomodulatory effect; USP21 can also hydrolyzes the K27/63-linked polyubiquitin chain on STING to negatively regulate DNA virus-induced type I interferon production [11].

Although Tregs may play an essential role in the regulation of the immune response during schistosome infection, the molecular mechanisms are not yet precisely defined. Therefore, this study is aimed at exploring the mechanism of action of USP21 in the regulation of the immune response during schistosome infection using the USP21 gene knockout mouse model. This study will describe the regulatory role of USP21 in the USP21 knockout mice infected with *S. japonicum* and related immune responses; we will further study the molecular mechanism underlying the effect of USP21 on the immune response to *S. japonicum*.

## 2. Materials and Methods

### 2.1. Ethics Statement

We carried out all the animal experiments in strict accordance with the Laboratory Animal Regulation (1988.11.1) and made every effort to minimize suffering. The IACUC of the National Institution of Parasitic Diseases of Chinese Center for Diseases Control and Prevention and Control approved all the procedures related to the use of experimental animals (License No. NJMU 07-0137). We used humane endpoints in this study. Due to the needs of the experiment, all experimental animals were euthanized on the 42^nd^ day after infection. We monitored the health of the animals daily. Tissues were harvested after animals were euthanized by CO_2_ exposure, which was confirmed by decapitation (to minimize the animal's suffering and distress). No animals died unexpectedly in this study. These animals were housed in groups of two to three per cage with free access to food and water on a 12 h light/dark cycle. Every effort was made to minimize the number and suffering of animals. The number of mice used in each test is listed in Table S1.

### 2.2. Experimental Animals, Parasites, and Establishment of Infection Models

Two strains of female mice, FOXP3^Cre^ (WT) and USP21^fl/fl^FOXP3^Cre^ (KO), were provided by Shanghai Institute of Bioscience, Chinese Academy of Sciences. The *S. japonicum* cercariae (Chinese mainland strain) were released from naturally infected *Oncomelania hupehensis* snails from the National Institute of Parasitic Diseases of Chinese Center for Diseases Control and Prevention (Shanghai, China). Mice from the two strains were divided into a normal control (NC/uninfected) group (*n* = 2) and an infection (INF) group (*n* = 10 ± 2) and were housed under specific pathogen-free conditions. All experimental animals were used at 6 to 8 weeks of age. Then, the mice in the INF group were infected with 25 ± 2 cercariae through the shaved abdominal skin. All experimental animals were sacrificed on the 42^nd^ day after infection [12]. The animals were included in the study if they were successfully infected with *S. japonicum*. If the animal died prematurely, follow-up experiment data were unable to be collected. Fifty-six animals were ultimately included in this study, and all met our inclusion and exclusion criteria. Serum, which was stored at -80°C and used for ELISAs, was separated from mouse blood that was collected from the tail vein at different times, namely, before infection, 20 days and 30 days after infection, and 42 days after infection. Blood was collected by eyeball enucleation. The animal experiments were conducted strictly with blinded protocols according to the Laboratory Animal Regulation, and we made every effort to minimize pain. The animals' cages were placed on the same shelf, and the feeding conditions and environment were as controlled as much as possible. For each animal, four different investigators were involved as follows: a first investigator (ZZP or QZQ) assigned the group based on the randomization table. This investigator was the only person aware of the group allocation. A second investigator (ZS, CSM, or HQ) was responsible for the normal feeding stage, whereas a third investigator (ZYX, LYY, XGN or XDH) performed the experimental procedures. Finally, a fourth investigator (LB, GJY, ZBX, ZFS or LY) (also unaware of treatment) assessed all experimental data. The mouse model of *S. japonicum* infection was repeated three times.

### 2.3. Collection of S. japonicum Worms and Measurement of the Weight of the Spleen and Liver of the Infected Mice

The mice were sacrificed by the cervical dislocation method 42 days after infection. The worms were collected from the portal vein by a cardiac infusion of saline, and the numbers of male and female adult worms and worm pairs were determined [12]. The spleens and livers of the mice were weighed and photographed.

### 2.4. HE and Masson Trichrome Staining

HE staining and Masson's trichrome staining have been previously described [13]. The remaining right liver lobe of each mouse was fixed with 4% paraformaldehyde and embedded in a paraffin block. We stained a section (5 *μ*m thick) with hematoxylin-eosin and performed the analysis with an inverted microscope (magnification 100x). The areas of single liver egg granulomas (*n* ≥ 5 per mouse) were measured using ImageJ software (NIH, Bethesda, USA), and the average number of eggs in a randomly selected visual field (*n* ≥ 5 per mouse) was counted at 40x magnification to evaluate the pathological changes and the number of eggs in the groups. Similarly, the same site of the liver from each mouse was stained with Masson's trichrome. We measured and analyzed the ratio of the collagen area (*n* ≥ 5 per mouse) using ImageJ software to evaluate the degree of hepatic fibrosis in the two groups.

### 2.5. Isolation of Peripheral Blood Mononuclear Cells (PBMCs) and Culture *In Vitro*

The cell preparation and cell culture methods have been previously described [14]. Blood (0.5-1 ml) was collected from the mice following eyeball enucleation at 42 days after infection. The serum and plasma were separated, and the serum was stored at -80°C until analysis. We mixed the plasma with sterile PBS at a ratio of 1 : 1, and the mixture was gently inverted approximately ten times. We then gently added the same volume of lymphocyte separation solution to form stratified layers. After centrifugation, we collected the middle layer, added red blood cell lysis buffer, and conducted another centrifugation step to obtain PBMCs. PBMCs were resuspended in complete culture medium containing 5 *μ*g/ml ConA to stimulate lymphocyte proliferation and maintain cell activity. The cell density, as determined under a microscope, was adjusted to 2 × 10^6^ cells/ml, and the cells were seeded into culture plates or dishes and cultured at 37°C with 5% CO_2_ for 72 h under sterile conditions. We then stored the culture supernatant after centrifugation at -80°C.

### 2.6. Spleen Cell Isolation and Culture *In Vitro*

Forty-two days after the infection, the mice were sacrificed by cervical dislocation method and the spleen was harvested [12]. The spleen was passed through a 100 *μ*m cell strainer to obtain a tissue homogenate, and red blood cell lysis buffer was repeatedly added until no red blood cells remained. Then, complete culture medium composed of RPMI 1640 medium supplemented with 10% fetal bovine serum (FBS) (GIBCO) and 1% Pen/Strep (GIBCO) was added to resuspend the cells. The cell density, as determined under a microscope, was adjusted to 2 × 10^5^ − 10^6^ cells/ml, and the cells were seeded in culture plates or dishes and cultured with 5 *μ*g/ml ConA to stimulate the cells at 37°C with 5% CO_2_ for 72 h under sterile conditions. We then stored the culture supernatant after centrifugation at -80°C.

### 2.7. Flow Cytometry

The flow cytometry method has been previously described [9]. For the cell surface marker and Tregs analysis, lymphocytes were isolated from the spleen, and the CD4^+^ T cells were enriched with CD4 (L3T4) MicroBeads mouse isolation kits (Miltenyi Biotec Inc., Auburn, CA 95602, USA). For the analysis of surface markers, the CD4^+^ T cells were stained with APC-conjugated anti-mouse CD4 (GK1.5, 1 : 100), PE-conjugated anti-mouse CD25 (PC61.5, 1 : 100), and FITC-conjugated anti-mouse FOXP3 (3G3, 1 : 100) antibodies from Tonbo Biosciences in PBS containing 2% FBS. The cell membranes were permeabilized, and the nuclear transcription factor FOXP3 was stained using the Transcription Factor Staining Buffer Kit (Tonbo Biosciences, San Diego, CA, USA) according to the manufacturer's instructions. Compensation was performed with a BD LSRFortessa (BD Biosciences). The data were acquired and analyzed with FlowJo software (Tree Star, Ashland, OR, USA).

### 2.8. RNA Extraction and RT-PCR

The RNA extraction and RT-PCR have been previously described [15]. We extracted the total RNA from 30 mg of liver and spleen tissues with TRIzol according to the manual. The quality and quantity of the RNA were then evaluated using an ultramicronucleic acid protease analyzer. The total RNA (1 *μ*g) was reverse transcribed with a RevertAid First-Strand cDNA Synthesis Kit (K1622, Thermo Fisher Scientific). The expression of USP21, IL-10, IL-17, and FOXP3 in the liver and spleen and a-Smooth muscle actin (*α*-SMA), collagen, and Collagen III in the liver was detected using the iTaq™ Universal SYBR® Green super mixture (1725121, Bio-Rad) and a fluorescence quantitative PCR 7500 system (Applied Biosystems, USA). The data were quantitatively analyzed using the 2^-*ΔΔ*Ct^ method with glyceraldehyde-3-phosphate dehydrogenase (GAPDH) as a control. The PCR cycling conditions were as follows: 40 cycles of 95°C for 30 s, 95°C for 5 s, and 60°C for 34 s, followed by 95°C for 15 s, 60°C for 60 s, 95°C for 15 s, and 60°C for 60 s. All the primers were subjected to a blast search with NCBI to ensure their specificity. All the primers are shown in Table 1.

### 2.9. Western Blotting

The Western blotting procedure has been previously described [16]. Liver tissue (30-50 mg) from each mouse was lysed on ice in cold RIPA lysis buffer (1-1.2 ml) containing a protease inhibitor cocktail (B14001, Bimake) for 3 to 4 h. After centrifugation at 14000 × g/min for 15 min, the supernatant was transferred to another tube to determine the total protein concentration using the BCA protein detection kit (P0010, Beyotime, China). The protein samples were boiled for 3 min, loaded onto a 10% polyacrylamide gel, electrophoresed, and transferred to a PVDF membrane (Millipore MA, USA). The proteins were separated using SDS–gel electrophoresis. The protein bands were detected by incubating the membrane with polyclonal primary antibodies against GAPDH (sc-32233, Santa Cruz), USP21 (ab171028, Abcam), *α*-SMA (no. 19245T, Cell Signaling Technology), Collagen I (BA0325, Boster), and Collagen III (sc-271249, Santa Cruz) for 12-16 h at 4°C, followed by an incubation with HRP-goat anti-rabbit IgG (ab97080, Abcam) and HRP-rabbit anti-mouse IgG (ab6728, Abcam) secondary antibodies. The immune complexes were visualized with the WesternBright™ ECL substrate (K-12045-D10, Advansta), and the luminescent signal was recorded with a chemiluminescence imaging system (Bio-Rad, USA) to determine the expression of the proteins. The expression of USP21, *α*-SMA, Collagen I, and Collagen III was quantified using GAPDH as a reference for relative quantification. ImageJ analysis software was applied to calculate the grayscale values of the bands on the images, which were used for statistical analysis (NIH, Bethesda, USA).

### 2.10. ELISA

The ELISA technique has been previously described [17]. Briefly, for the *S. japonicum*-SEA and *S. japonicum*-SWAP ELISAs, all the native antigens were diluted to a final concentration of 1 *μ*g/ml with coating buffer; for the *S. japonicum*-SEA and *S. japonicum*-SWAP ELISAs, 50 ng of each antigen in 100 *μ*l was added to each well and incubated at 4°C overnight. After blocking with blocking buffer (1% BSA in PBST) at 37°C for one hour, the serum samples diluted 1 : 250 with blocking buffer were added (100 *μ*l/well) and incubated for one hour at 37°C. HRP-goat anti-mouse IgM (ab97230, Abcam) and HRP-rabbit anti-mouse IgG (ab6728, Abcam) were used as the secondary antibodies (1 : 20000, 100 *μ*l/well), and the samples were incubated for one hour at 37°C. Streptavidin-HRP (BD Pharmingen, CA, USA) (1 : 10000) was then applied to each well (100 *μ*l/well). The wells were washed with PBST five times for 2 min each after each step. The reactions were developed using TMB as a substrate (100 *μ*l/well) for 5 min, and this development was stopped using 2 M sodium hydroxide (50 *μ*l/well). The optical density (OD) values were recorded at 450 nm using a microplate reader, and all the tests were performed in duplicate on each test plate.

### 2.11. Cytokine Detection (Multiplex Fluorescent Microsphere Immunoassay)

The mouse serum and cell culture samples were prepared according to the manufacturer's instructions (Laizee Biotech Co. Ltd., ppx-6, China). Fifty microliters of premixed beads was added to each of the 96 wells and then incubated with the samples. The detection antibodies (IFN-*γ*, IL-4, IL-10, IL-17A, IL-23, and IL-9) were added after an initial wash, followed by a second wash and the addition of SA-PE. The clean and dried 96-well plate was placed in the Bio-Plex 200 instrument for detection. The standard curve was fitted using a five-parameter nonlinear regression method, and the concentration was calculated. The results included the label, the median of the fluorescence intensity, and the concentration [18].

### 2.12. Statistical Analyses

Statistical comparisons were performed with Prism 7.0 (GraphPad Prism.) and SPSS 18.0 software using a *t*-test for comparisons of two datasets and ANOVA for multiple comparisons. All the data are presented as the $\bar{x}\pm \text{S}.\text{D}.$, and the differences were considered statistically significant at *p* ≤ 0.05.

## 3. Results

### 3.1. Depletion of USP21 in Treg Cells Weakens the Resistance to *S. japonicum* in Infected Mice

**Figure 1:**
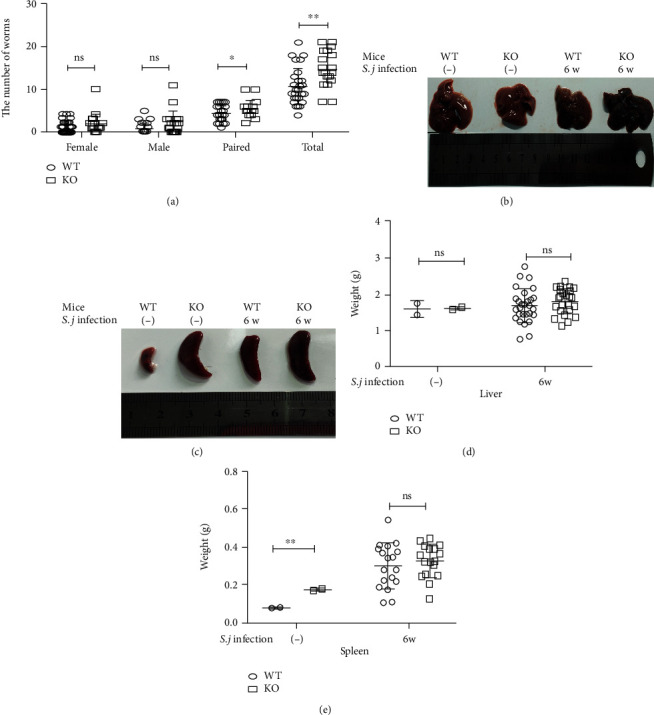
Deletion of USP21 in Tregs reduces the resistance to *S. japonicum* in infected mice. (a–e) FOXP3^Cre^ (WT) and USP21^fl/fl^FOXP3^Cre^ (KO) mice (*n* = 26 ± 2/group) were infected with 25 ± 2 cercariae through the abdomen and were sacrificed 6 weeks after infection. The liver and spleen tissues were collected, and the adults were recovered. (a) Comparison of the number of females, males, pairs, and adults recovered from the WT- and KO-infected (INF) groups (*n* = mean ± SD), ^∗^*p* = 0.01 for the total number of adults and ^∗∗^*p* = 0.036 for the total number of worm pairs. (b) Representative images of the livers of the WT and KO mice from the NC and INF groups. (c) Liver weight of the WT and KO mice in the NC and INF groups (weight = mean ± SD); *p* > 0.05 was considered a difference that was not statistically significant between the normal control group and the infected group. (d) Representative images of the spleens of the WT and KO mice in the NC and INF groups. (e) Spleen weight of the WT and KO mice in the NC and INF groups (weight = mean ± SD); ^∗∗^*p* = 0.007 compared with the normal control group; *p* > 0.05 represented a difference that was not statistically significant compared with the infected group.

Both WT and KO mice were infected with *S. japonicum*, and the adult parasites were recovered (according to the steps described below) to observe the differences between the mouse strains. The number of total adult parasites (females and males combined) recovered from KO mice was significantly greater than the number recovered (Figure 1(a)). However, no difference was found in the development of cercariae between male and female adult parasites. The numbers of single males, single females, and pairs and the total number of adults were counted. We weighed the liver and spleen of the mice and observed changes in their color, shape, and texture. The color and size of the liver and spleen of the KO-INF group were slightly darker and larger than the WT group, and the KO mice suffer from splenomegaly even if they were uninfected (Figures 1(b)–1(e)).

### 3.2. Effect of USP21 on *S. japonicum* Eggs during Infection in KO Mice

**Figure 2:**
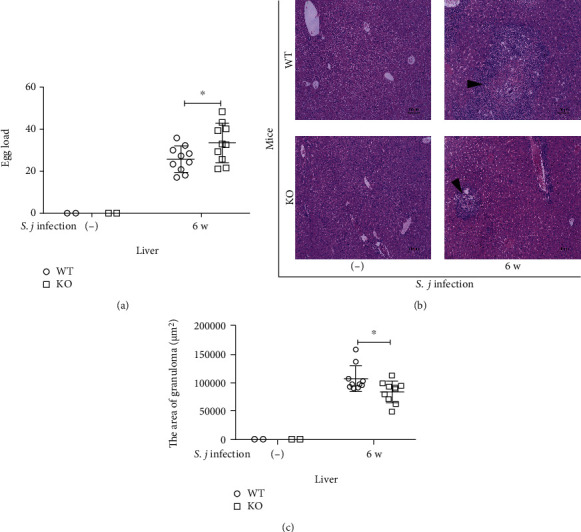
Effect of USP21 on *S. japonicum* eggs in infected KO mice. (a–c) The livers were harvested from the mice on the 42^nd^ day after the infection for HE staining, and the number of eggs was counted under the microscope (*n* = 2 from the uninfected group and *n* = 10 from the infected group). (a) Statistical graph of the eggs in the livers of the WT-INF group and the KO-INF group (the data are presented as the means ± SD, ^∗^*p* = 0.043). (b) Representative images of HE staining in the livers of WT-INF and KO-INF groups (original magnification: 100x). The arrows indicate the granulomas. (c) Comparison of the liver egg granuloma areas between the WT-INF and KO-INF groups. The data are presented as the means ± SD, ^∗^*p* = 0.022.

A portion of the liver obtained from the mice 42 days after infection was used for HE staining to observe the pathological changes in egg granuloma formation. We analyzed the granuloma area and egg number in the KO mice and WT mice using ImageJ software. A significantly greater number of eggs was observed in the KO mice than in the WT mice (Figure 2(a)), but the number of inflammatory cells in the egg granulomas of the KO mice was lower than in the WT mice, and lighter HE staining was observed in the KO mice (Figure 2(b)). The areas of the liver egg granulomas in the KO mice were significantly smaller than those in the WT mice (Figure 2(c)).

### 3.3. Changes in Liver Fibrosis in USP21^fl/fl^FOXP3^cre^ Mice Infected with *S. japonicum*

**Figure 3:**
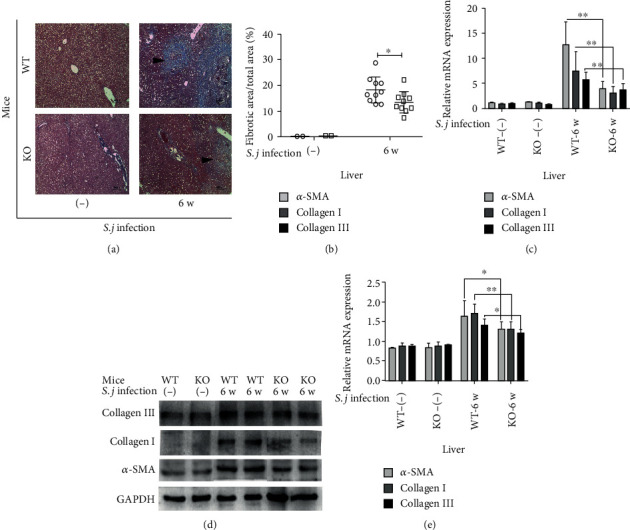
Changes in liver fibrosis in USP21^fl/fl^FOXP3^cre^ mice infected with *S. japonicum*. (a–e) The livers were harvested from the mice on the 42^nd^ day after the infection and stained with Masson's trichrome (*n* = 10 animals/group). (a) Representative images of collagen deposition in the WT-INF and KO-INF mice (original magnification: 100x) were obtained. The blue areas are collagen granules. (b) Comparison of the percentages of the hepatic fibrosis area between the WT-INF and KO-INF groups. The data are presented as the means ± SD, ^∗^*p* = 0.039. (c) Comparison of the levels the *α*-SMA, Collagen I, and Collagen III mRNAs in the WT and KO mice in the NC and INF groups. The data are presented as the means ± SD. Comparison of the levels of the *α*-SMA, Collagen I, and Collagen III mRNAs between the infected groups. The data are presented as the means ± SD. In the infection group, *α*-SMA: ^∗∗^*p* < 0.001; collagen I: ^∗∗^*p* = 0.008, and Collagen III: ^∗∗^*p* = 0.006. (d) Representative images of SMA, Collagen I, and Collagen III protein expression in the WT and KO mice from the NC and INF groups, as determined by Western blotting. (e) Comparison of protein expression between WT and KO mice in the NC and INF groups. The data are presented as the means ± SD. In the infection group, *α*-SMA: ^∗^*p* = 0.034, Collagen I: ^∗∗^*p* = 0.006, and Collagen III: ^∗^*p* = 0.03.

The expansion of collagen fibers, which reflects the severity of hepatic fibrosis, was observed using Masson's trichrome staining (Figure 3(a)). *α*-SMA is a hallmark of activated myofibroblasts and has been extensively used to indicate the occurrence and severity of fibrosis in individuals with liver diseases [13]. In hepatic fibrosis associated with schistosomiasis, the expression level of *α*-SMA is elevated [13]. Therefore, the expression of the *α*-SMA, Collagen I, and Collagen III mRNAs was detected using RT-PCR, and the protein expression of these factors was analyzed using Western blotting. Fewer collagen fibers with a lighter staining intensity were observed in the KO mice than in the WT mice, and these differences were statistically significant (Figure 3(b)). Significantly lower levels of the *α*-SMA, Collagen I, and Collagen III mRNAs and proteins were detected in the KO mice than in the WT mice (Figures 3(c)–3(e)).

### 3.4. Hepatic Immunity in USP21^fl/fl^FOXP3^cre^ Mice Infected with *S. japonicum*

**Figure 4:**
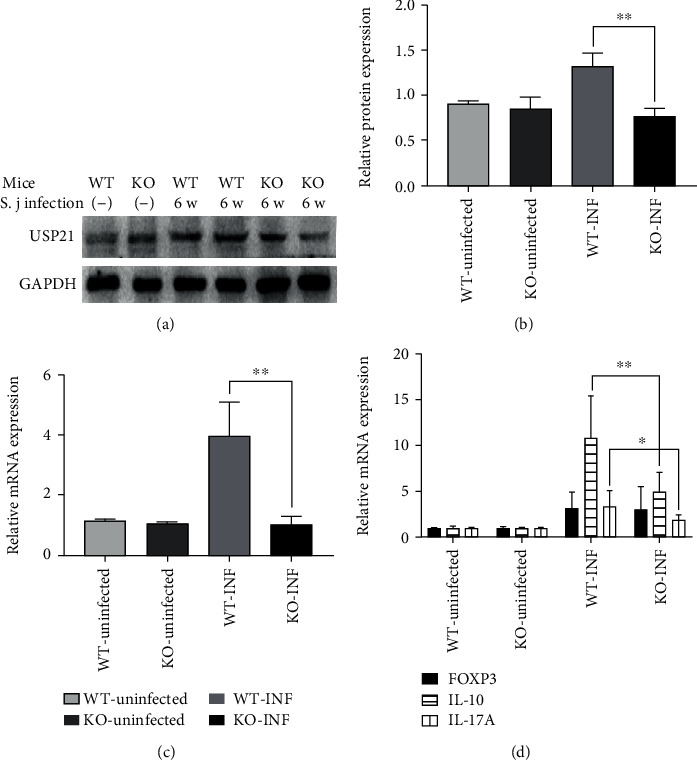
Liver immunity in USP21^fl/fl^FOXP3^cre^ mice infected with *S. japonicum*. (a) Representative images of USP21 protein expression in the in WT and KO mice in the NC and INF groups determined using Western blotting (*n* = 6 mice/group). (b) Comparison of protein expression in the WT and KO mice in the NC and INF groups (*n* = 6 mice/group). The data are presented as the means ± SD, and the difference between the infection groups was significant (^∗∗^*p* < 0.001). (c) Comparison of the USP21 mRNA levels between WT and KO mice in the NC and INF groups (*n* = 6 mice/group). The data are presented as the means ± SD, ^∗∗^*p* = 0.001 for the comparison between the infection groups. (d) Comparison of the levels of the FOXP3, IL-10, and IL-17 mRNAs in the WT and KO mice from the NC and INF groups (*n* = 6 mice/group). The data are presented as the means ± SD; for the comparison between the infection groups: IL-10 ^∗∗^*p* = 0.003 and IL-17 ^∗^*p* = 0.026.

We detected the expression of the IL-10, IL-17, and FOXP3 mRNAs in the liver to determine whether the immune status of the liver was consistent with the condition of the spleen. We also detected the expression levels of the USP21 protein and mRNA. Lower expression levels of the USP21 protein and mRNA were detected in the liver of KO mice than in WT mice (Figures 4(a)–4(c)). Significantly lower expression of the IL-10 and IL-17 mRNAs was observed in the KO mice than in the WT mice (Figure 4(d)).

### 3.5. Changes in Spleen Immunity in USP21^fl/fl^FOXP3^cre^ Mice Infected with *S. japonicum*

**Figure 5:**
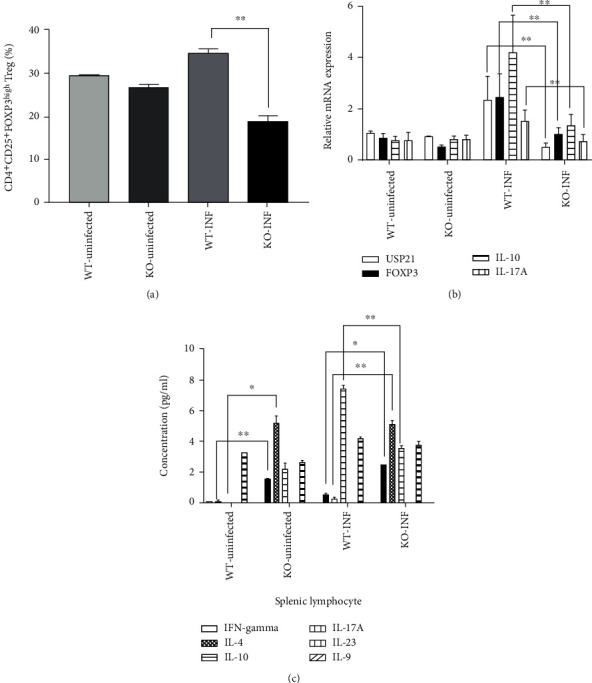
Changes in splenic immunity in USP21^fl/fl^FOXP3^cre^ mice infected with *S. japonicum*. (a–c) The spleens were harvested from the mice on the 42nd day after the infection. (a) CD4^+^CD25^+^FOXP3^high^ cells directly isolated from the spleens of WT and KO mice in the NC and INF groups were identified using flow cytometry at 42 days after the infection with *S. japonicum*. Comparison of the proportion of CD4^+^CD25^+^FOXP3^high^ cells among the CD4^+^ T cell population in the WT and KO mice in the NC and INF groups. The data are presented as the means ± SD, infection groups: ^∗∗^*p* = 0.007. (b) Comparison of the mRNA levels of FOXP3, IL-10, IL-17, and USP21 in the WT and KO mice in the NC and INF groups, and the data are presented as the means ± SD. For the comparison between the infection groups, FOXP3 ^∗∗^*p* = 0.001, IL-10 ^∗∗^*p* < 0.001, IL-17 ^∗∗^*p* < 0.001, and USP21 ^∗∗^*p* < 0.001. (c) The splenic cells were cultured *in vitro* (*n* = 6/group). Comparison of the levels of IFN-gamma, IL-4, IL-10, IL-17A, IL-23, and IL-9 in cultured spleen cells from the WT and KO mice in the NC and INF groups. The data are presented as the means ± SD. In the NC group, IFN-gamma ^∗∗^*p* = 0.002 and IL-4 ^∗^*p* = 0.002; for the comparison between the infection groups, IFN-gamma ^∗^*p* = 0.013, IL-4 ^∗∗^*p* = 0.005, and IL-10 ^∗∗^*p* = 0.004.

In an attempt to understand the differences in the splenic immune cells between the KO and WT mice after infection with *S. japonicum*, splenic cells from the two groups, including the NC group, were collected, isolated, and cultured *in vitro* to detect and analyze the number of Tregs and the percentage of CD4^+^CD25^+^FOXP3^high^ cells among the CD4^+^ cell population. The comparison of the proportion of CD4^+^CD25^+^FOXP3^high^ cells among the CD4^+^ T cell population in the WT and KO mice from the NC and INF groups is shown in Figure 5(a). The percentage of FOXP3^high^Tregs in the KO-uninfected group and WT-uninfected group was not significantly different, while the percentage of FOXP3^high^Tregs in the KO-INF group was significantly lower than in the WT-INF group. The changes in the types of T cells were revealed by detecting the relative mRNA expression of IL-10, IL-17, FOXP3, and USP21 in the spleen using RT-PCR and by the detection of the levels of IFN-gamma, IL-4, IL-10, IL-17A, IL-23, and IL-9 in the splenocytes using a multiplex fluorescent microsphere immunoassay. Lower levels of the IL-10, IL-17, FOXP3, and USP21 mRNAs were detected in the KO mice than in the WT mice (Figure 5(b)). The results of splenic cell culture cytokine production showed significantly higher levels of IFN-gamma and IL-4 in the KO-uninfected group than in the WT-uninfected group. In the comparison between the two INF groups, the IFN-gamma and IL-4 contents in KO mice were also higher than in WT mice, and the content of lL-10 was higher in WT mice (Figure 5(c)).

### 3.6. Specific Antibody Responses in USP21^fl/fl^FOXP3^Cre^ Mice Infected with *S. japonicum*

Serum samples were collected from the KO and WT mice to measure the contents of anti-SEA and anti-SWAP IgG/IgM antibodies at different stages of the infection and to better understand the specific antibody responses of USP21^fl/fl^FOXP3^Cre^ mice to *S. japonicum*.

**Figure 6:**
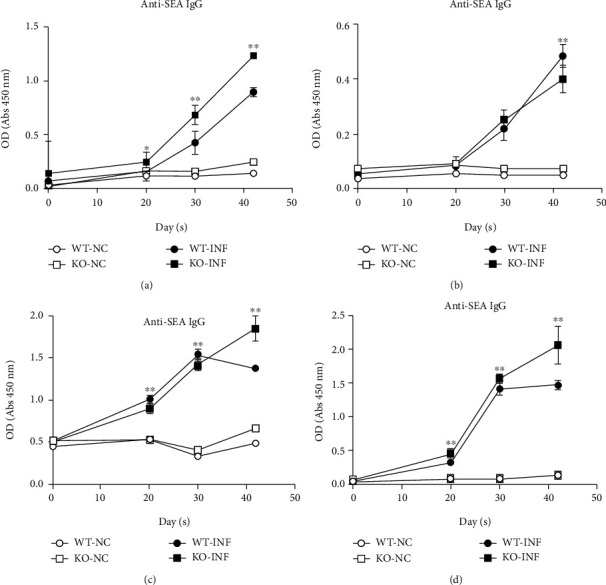
Specific antibody responses in USP21^fl/fl^FOXP3^Cre^ mice infected with *S. japonicum*. (a–d) Serum collected from the WT and KO mice in the NC and INF groups (*n* = 26 ± 2/group) at different stages of the infection (uninfected, day 20, day 30, day 42). (a) Comparison of the changes in the anti-SEA IgG content. The data are presented as the means ± SD. For the comparison between infection groups, ^∗^*p* = 0.01 on day 20, ^∗∗^*p* < 0.001 on day 30, and^∗∗^*p* < 0.001 on day 42. (b) Comparison of the anti-SEA IgM content. The data are presented as the means ± SD, and ^∗∗^*p* = 0.002 on day 42 when comparing between the infection groups. (c) Comparison of the anti-SWAP IgG contents. The data are presented as the means ± SD; for the comparison between infection groups, ^∗∗^*p* < 0.001 on day 20, ^∗∗^*p* = 0.003 on day 30, and ^∗∗^*p* < 0.001 on day 42. (d) Comparison of the anti-SWAP IgM contents. The data are presented as the means ± SD. For the comparison between infection groups, ^∗∗^*p* < 0.001 on day 20, ^∗∗^*p* = 0.004 on day 30, and ^∗∗^*p* < 0.001 on day 42.

The analysis of the anti-SEA content revealed a higher level of anti-SEA IgG secretion in the KO-INF group than in the WT-INF group at all stages of infection, and the trends of the two groups were consistent and increased (Figure 6(a)). Meanwhile, the level of anti-SEA IgM secretion in the KO-INF group was lower than that in the WT-INF group beginning on the 30^th^ day after *S. japonicum* infection to the 42^nd^ day, and the trend of the changes in the two groups was basically the same (Figure 6(b)).

The anti-SWAP IgG level in the WT-INF group decreased beginning on the 30^th^ day, and the level of IgG secretion in the preceding 30 days was higher than in the KO-INF group. The level of secreted IgG in the KO-INF group increased until the 42^nd^ day after *S. japonicum* infection (Figure 6(c)). Based on the graph showing the change in the secretion of anti-SWAP IgM, the level in the KO-INF also increased until the 42^nd^ day after *S. japonicum* infection and was always higher than the level in the WT-INF group. However, the anti-SWAP IgM level in the WT-INF group remained basically unchanged after the 30^th^ day (Figure 6(d)).

### 3.7. Serum Cytokine Levels in USP21^fl/fl^FOXP3^Cre^ Mice Infected with *S. japonicum*

We measured the levels of IFN-gamma, IL-4, IL-10, IL-17A, IL-23, and IL-9 in the serum samples and PBMCs from the KO and WT mice at different stages of infection to conduct and extensive analysis of how USP21-deficient Tregs affect the resistance of mice to *S. japonicum*.

**Figure 7:**
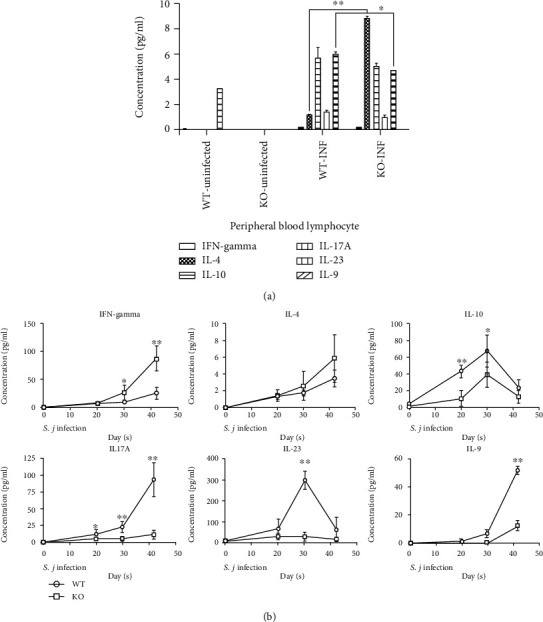
Serum cytokines in USP21^fl/fl^FOXP3^Cre^ mice infected with *S. japonicum*. (a) Comparison of the concentrations of IFN-gamma, IL-4, IL-10, IL-17A, IL-23, and IL-9 in the cultures of peripheral blood lymphocytes isolated from the WT and KO mice in the NC and INF groups 42 days after the infection (*n* = 10 mice/group). The data are presented as the means ± SD, IL-4 ^∗∗^*p* = 0.008, and IL-23 ^∗^*p* = 0.035 for the comparison between the infection groups. (b) Comparison of the serum IFN-gamma, IL-4, IL-10, IL-17A, IL-23, and IL-9 levels in the WT- and KO-INF groups (*n* = 26 ± 2/group) at different time points (uninfected, 20^th^ day after infection, 30^th^ day after infection, and 42^nd^ day after infection). The data are presented as the means ± SD. IFN-*γ*^∗^*p* = 0.024 on day 20 and ^∗∗^*p* < 0.001 on day 42; IL-10 ^∗∗^*p* < 0.001 on day 20 and ^∗^*p* = 0.017 on day 30; IL-17A ^∗^*p* = 0.026 on day 20, ^∗^*p* < 0.001 on day 30, and ^∗∗^*p* < 0.001 on day 42; IL-23 ^∗∗^*p* < 0.001 on day 30; and IL-9 ^∗∗^*p* < 0.001 on day 42.

In the peripheral blood lymphocyte culture, the concentration of each cytokine in the KO-uninfected group was basically the same as the WT-uninfected group. The IL-4 level was significantly increased in the KO-INF group compared with the WT-INF group, whereas a lower IL-23 level was detected in the KO-INF group than in the WT-INF group. The concentrations of other cytokines in the KO-INF group were higher than those in the WT-INF group, but the differences were not statistically significant (Figure 7(a)).

Serum measurements performed at different stages of the *S. japonicum* infection revealed similar trends for the changes in the IFN-gamma, IL-4, IL-10, IL-17A, and IL-9 levels in the WT-INF and KO-INF groups. The levels of secreted IFN-gamma and IL-4 in the WT-INF and KO-INF groups increased, and the level in the KO-INF group was significantly higher than that in the WT-INF group. Although the levels of IL-17A and IL-9 increased, the level in the KO-INF group was significantly lower than that in the WT-INF group. The level of secreted IL-10 in the two groups decreased on the 30^th^ day, and the level secreted in the KO-INF group was always lower than that in the WT-INF group. Interestingly, the levels of IL-23 differed between the WT-INF and KO-INF group. The level in the KO-INF group remained basically unchanged, while the level in the WT-INF group increased from day 0 to day 30 of infection, and its concentration decreased rapidly from day 30 to day 42, with statistically significant differences (Figure 7(b)).

## 4. Discussion

In mammalian hosts, *S. japonicum* stimulates immune responses during all stages of its life cycle, which are activated by Th1, Th2, and Th17 cells [19]. Meanwhile, Tregs play an essential immunomodulatory role in curbing overactive T cell responses [20, 21]. USP21 is a member of the ubiquitin-specific proteolytic enzyme (USP) family that exerts different biological functions. For example, USP21 regulates gene expression in hepatocytes during liver regeneration by catalyzing the hydrolysis of ubH2A [22]. During inflammation, USP21 negatively regulates RIPK1 to inhibit its activity downstream of TNFR1 [23], and the USP21-mediated deubiquitination of IL-33 promotes the transcription of NF-*κ*B p65 [24]. Additionally, USP21 binds to and deubiquitinates RIG-I in the cytoplasm to play an immunomodulatory role in antiviral responses [25]. More recently, USP21 was shown to regulate the expression of factors related to cell cycle, proliferation, and craniofacial development by deubiquitinating FOXM1 and goosecoid (GSC) [26, 27].

Previously, researchers used a USP21 knockout mouse model to explore its biological role in the differentiation of lymphocytes and hematopoietic stem cells [28]. They found that elderly USP21 knockout mice exhibited spontaneous T cell activation and splenomegaly. Furthermore, another study showed that the depletion of USP21 induces the production of Th1-like Tregs as a result of unstable FOXP3 expression, leading to severe autoimmune systemic disorders [9]. Thus, we used the model of USP21 knockout mice with *S. japonicum* infection to illustrate the role of unstable Tregs in *S. japonicum* infection.

Schistosome infection can be divided into three stages, namely, acute infection, active infection, and late chronic infection, and these stages present different clinical symptoms and immune mechanisms [2]. Differences in clinical symptoms and immune mechanisms of these stages have been identified. In the present study, after *S. japonicum* infection, there was a greater number of pairs and adult parasites recovered from the KO mice than that from the WT mice, and a similar trend was noted for the number of eggs recovered. In addition, no difference was observed between the female and male adults recovered from the two groups. *Schistosoma* depends on host signals, such as TGF-*β* and TNF-*α*, to maintain its proper development and maturation [29, 30]. We speculated that the changes in the immune microenvironment in the host were potentially caused by the absence of USP21 and Treg dysfunction, which might promote the development of cercariae and the male and female combination of *S. japonicum*. Considering that protection from *S. japonicum* infection mainly depends on the clearance of *S. japonicum* in the early stage of infection, USP21^−/−^ Tregs might inhibit the ability of mice to cause the death of *S. japonicum*. These two theories require further evaluation in the future.

According to recent studies, liver egg granuloma and fibrosis are the main pathogenic pathological responses, and the severity of these symptoms is usually related to the intensity of infection [2]. The major mechanism of liver fibrosis is the abnormal expression of collagen and the excessive deposition of ECM (Extracellular matrix) caused by the activation of HSCs (hepatic stellate cells). Normally, the HSCs are mainly distributed in the hepatic sinusoid space to store vitamin A in a resting state [31, 32]. When chronic liver damage occurs, resting HSCs are activated and converted into myofibroblasts that then secrete a large amount of ECM and many fibrogenic cytokines, such as *α*-SMA and transforming growth factor-*β*1 (TGF-*β*1) [33, 34]. The deposition of ECM containing collagen fibers leads to the imbalance of ECM degradation and the synthesis, eventually leading to damage to the liver structure and liver fibrosis [35]. Studies have confirmed that HSC activation critically regulates the development of liver fibrosis, and HSC inhibition improves the process of liver fibrosis, thereby reducing the degree of liver fibrosis.

In our study, the diameter of the egg granulomas in the KO mice was smaller than that of the egg granulomas in the WT mice at six weeks, the levels of proinflammatory cytokines/chemokines and infiltrating neutrophils were reduced, and the HE staining was lighter. The degree of hepatic fibrosis in the KO mice was also lower than that in the WT mice, as determined using RT-PCR, Western blotting, and Masson's trichrome staining. Therefore, USP21-deficient Tregs might inhibit HSC overactivation and further reduce pathological liver damage. The liver fibrosis and splenic diseases caused by *S. japonicum* are associated with the great number of FOXP3^+^ Tregs in the blood [7]. According to previous studies, Th17 cells might play an essential role in the immunopathology in the liver and the formation of egg granulomas, and the development of severe schistosomiasis in mice is associated with high levels of IL-17A [36, 37]. IL-17-deficient mice showed higher IFN-*γ* levels and reduced immunopathology [38], similar to the USP21 KO mice with downregulated IL-17,increased IFN-*γ* and reduced immunopathology. The results from this previous study further suggested that IL-17 may affect these changes in infected KO mice. The results of the present study also confirmed that the IL-17A in the KO group was lower than that in the WT group, but a similar trend was noted for the content of IL-10. Therefore, we speculated that the absence of USP21 might inhibit the Th17-type immune response, which exerted a positive effect on the reduction of liver immunopathological damage and might also lead to immune dysfunction in the host. The number of Tregs was decreased in KO mice; however, the hepatic immunopathology was suppressed. We speculated that upon *S. japonicum* infection, Tregs were overactivated in mice lacking USP21, although unstable Tregs lose their immunosuppressive effects, disrupting the host immune mechanism and weakening the related immune pathology caused by eggs. However, more in-depth researches are needed on the specific mechanism to determine which pathway affects liver immunopathology.

Additionally, the expression of the USP21 mRNA was detected in the liver and spleen. The decrease in USP21 expression in the spleen of KO mice might be associated with overactivation of Tregs. The absence of USP21 destabilized the expression of FOXP3 and led to increased T cells. The percentage of FOXP3^+^ Tregs was reduced in KO mice due to the degradation of FOXP3. Meanwhile, we speculated that unstable FOXP3 led to the generation of T helper-like Tregs. The higher expression of USP21 in liver of the WT mice was related to the excessive activation of Tregs. In the WT mice, liver inflammation and liver fibrosis were more serious, and the number of parasites recovered from tissues and cells was increased, and the hepatocyte activity and USP21 expression were increased.

Soluble egg antigen (SEA) and adult worm antigen (SWA) are the main soluble proteins that are targeted by the adaptive immune response induced by *S. japonicum* infection. Related studies have shown that a high level of anti-*Schistosoma* IgG is associated with increased susceptibility to parasites and that anti-*Schistosoma* IgG, especially the IgG4 response [39], is positively correlated with severe schistosomiasis. In this study, anti-SEA and anti-SWA IgG in the USP21 knockout mice infected with *S. japonicum* were higher than those in the WT mice, while the IgM levels were not significantly different between the two groups. Based on this finding, USP21-deficient Tregs increased the susceptibility of mice to schistosomiasis.

Previous studies have shown that the immunologic process of *S. japonicum* infection is an initial short-lived increase in the number IFN-*γ*-producing Th1 cells, resulting in a mostly host-protective, Th2- (IL-4, IL-5, and IL-13) dominated environment [40, 41]. Cytokines, such as IL-4, IL-10, IFN-*γ*, IL-17, and IL-9, have been reported to be key signals in the immune cells involved in schistosomiasis. IL-4, a typical type 2 immune factor, attenuates Tregs function in type 2 inflammatory diseases through IL-4 receptor *α* (IL-4R*α*) signaling. Increased IL-4R*α* levels, through mutations in their functional domains or chronic inflammation of type 2 immune responses, leads to a rapid reduction in the FOXP3^+^ Treg population and the impairment of Tregs function, driving the polarization of Tregs to Th2- or Th17-like cells [42, 43]. Other studies have shown that Tregs need IL-4R*α* to control inflammation during worm infection [44]. IL-10 has been reported to have dual functions, namely, proinflammatory and anti-inflammatory effects, which, on the one hand, might inhibit Th1-type autoimmune diseases and aggravate autoantibody-mediated autoimmune diseases, such as lupus erythematosus and myasthenia gravis, and, on the other hand, ameliorate parasite-mediated autoimmune disease [45]. Tregs are important components of the immunomodulatory network in chronic worm infection, especially the immunosuppression of chronic schistosomiasis, which is characterized by an independent downregulation of IL-10 produced by Th2 cytokines [46, 47].

Higher levels of IFN-*γ* and IL-4 were detected in the KO mice than those in the WT mice in the present study, while lower IL-10, IL-17A, IL-23, and IL-9 levels were observed in the KO mice than those in the WT mice. After different durations of infection, the trends of the changes in the serum cytokine levels in the two groups were approximately the same. These results are consistent with previous findings [9] and prompted us to speculate that the depletion of USP21 transforms Tregs to Th1-like cells. In the later stage of *S. japonicum* infection, IFN-*γ* levels remained stable, while IL-4 levels increased. However, this result was different from those mentioned above, which showed that the IL-4 was higher in the KO mice than that in the WT mice. The unstable Tregs might exhibit different differentiation patterns because of the different microenvironments present during schistosome infection. A series of T cell activation steps and long-term stimulation of chronic inflammatory factors might result in low reactivity, which was basically consistent with the results from the present study [9, 48]. This phenomenon was consistent with the reduced levels of secreted factors, in addition to IFN-*γ* and IL-4. The result also suggested that Th1 and Th2 cells are the main immune cell types involved in schistosomiasis. IL-23 is one of the driving factors that induce IL-17A expression [21]. We also observed that IL-17A was related to IL-23, both of which were downregulated and present at lower levels in the KO group than those in the WT group.

Worms live for a long time in the host, and accumulating evidence suggests that they can manipulate the host immune system through the host immunomodulatory network [47, 49]. As shown in our study, unstable Tregs in mice infected with *S. japonicum* might allow the host to benefit from the excessive inflammatory responses. This study still has some limitations. In particular, the eventual survival status of the mice was not considered.

In conclusion, USP21 is an important immunomodulatory molecule in the host that plays an important role in host immunity to *S. japonicum* infection. Our study might provide a theoretical basis for further analysis of the USP21-mediated regulation of different immune cell types activated by *S. japonicum*.

## Figures and Tables

**Table 1:** Primers used for the mRNA analysis.

Gene	Forward primer (5′→3′)	Reverse primer (5′→3′)
GAPDH	ACTCCACTCACGGCAAATTC	TCTCCATGGTGGTGAAGACA
USP21	ACCCAGGAAAGACAGCAACC	CTCGAAGACCTTCTCACAACCA
FOXP3	GTGATTTTAATAAGCTCCAAGACCA	GATCATCATGTATGCTTCTATGCAG
IL-10	TATCCCTCTGTGATCTGGGAAG	ATCTTCTCGACCCTGAAAGTGA
IL-17	CACAGCCCTGGTGTGCGACAAT	TTGCTCTGGGCTTCATCCCCCA
*α*-SMA	TCCTGCGCCTAATGTCCACCGA	AAGCGACTGTTGCCTTCGCCTC
Collagen I	TCCTGGTGGCAAGGGTGATCGT	TGGAGCACCAGAAGGACCAGCA
Collagen III	GCTCACCACACACTGCTTCT	GGATTCACAGCTTCACAGGA

## Data Availability

All relevant data are within the manuscript and its Supporting Information files.

## Supplementary Materials

Supplementary Materials Table S1: number of mice used in each test.
