# Supplementary material for: Schistosoma japonicum Infection in Treg-Specific USP21 Knockout Mice
Source: J Immunol Res. 2021 Feb 9;2021:6613162. doi: 10.1155/2021/6613162 (PMC7886505; doi:10.1155/2021/6613162)
Supplement: Supplementary Materials — Table S1: number of mice used in each test. [file 6613162.f1.pdf]

Table S1. Number of mice used in each test

ID	Test	Number of mice used	Figure number
1	Collection of S. japonicum worms and weight of the spleen and liver of the infected mice	N=28 for WT-INF,<br>N=24 for KO-INF,<br>N=2 for both WT-NC and KO-NC	Fig. 1
2	HE staining for the average number of eggs in liver and liver egg granuloma areas	N=10 for both WT-INF and KO-INF,<br>N=2 for both WT-NC and KO-NC	Fig. 2
3	Masson staining for tests the degree of hepatic fibrosis	N=10 for both WT-INF and KO-INF,<br>N=2 for both WT-NC and KO-NC	Fig. 3a and 3b
5	RT-PCR study for the expression of α-SMA, collagen-I and collagen-III (Liver)	N=10 for both WT-INF and KO-INF,<br>N=2 for both WT-NC and KO-NC	Fig. 3c
6	Western Blotting for the expression of α-SMA, collagen-I and collagen-III (Liver)	N=10 for both WT-INF and KO-INF,<br>N=2 for both WT-NC and KO-NC	Fig. 3d and 3e
7	Western Blotting for USP21 expression	N=6 for both WT-INF and KO-INF,<br>N=2 for both WT-NC and KO-NC	Fig. 4a and 4b
8	RT-PCR study for the expression of FOXP3, IL-10, IL-17 and USP21(Liver)	N=6 for both WT-INF and KO-INF,<br>N=2 for both WT-NC and KO-NC	Fig. 4c and 4d
9	Flow cytometry analysis	N=4 for both WT-INF and KO-INF,<br>N=2 for both WT-NC and KO-NC	Fig. 5a
10	RT-PCR study for the expression of FOXP3, IL-10, IL-17 and USP21 (Spleen)	N=10 for both WT-INF and KO-INF,<br>N=2 for both WT-NC and KO-NC	Fig. 5b
11	Multiplex Fluorescent Microsphere Immunoassay for tests the contents of IFN-gamma, IL-4, IL-10, IL-17A, IL-23 and IL-9 in cultured	N=6 for both WT-INF and KO-INF,<br>N=2 for both WT-NC and KO-NC	Fig. 5c

	spleen cells		
12	ELISA test (measure the content of anti-SEA and anti-SWAP IgG/IgM antibodies during the different stages of infection).	N=28 for WT-INF,<br>N=24 for KO-INF,<br>N=2 for both WT-NC and KO-NC	Fig. 6
13	Multiplex Fluorescent Microsphere Immunoassay for tests the contents of IFN-gamma, IL-4, IL-10, IL-17A, IL-23 and IL-9 in the cultures of peripheral blood lymphocytes	N=10 for both WT-INF and KO-INF,<br>N=2 for both WT-NC and KO-NC	Fig. 7a
14	Multiplex Fluorescent Microsphere Immunoassay for tests the contents of IFN-gamma, IL-4, IL-10, IL-17A, IL-23 and IL-9 in the serum at different times	N=28 for WT-INF,<br>N=24 for KO-INF,<br>N=2 for both WT-NC and KO-NC	Fig. 7b

WT, FOXP3^{Cre}mice; KO, USP21$^{fl/fl}$FOXP3^{Cre} mice.
